# Dexmedetomidine’s Role in Adult ICU After 20 Years of Experience—A Narrative Review

**DOI:** 10.3390/healthcare13222882

**Published:** 2025-11-12

**Authors:** Eleni N. Sertaridou, Maria Fountoulaki, Abhishek Jha, Vasilios E. Papaioannou, Christina Alexopoulou

**Affiliations:** 1 ICU Department, University Hospital of Alexandroupolis, 68100 Alexandroupolis, Greece; elenisertaridou@yahoo.com; 2 Second Department of Anaesthesiology, “Attikon” University Hospital, National and Kapodistrian University of Athens, 12461 Athens, Greece; mafountoulaki@gmail.com; 3 King’s College Hospital NHS Foundation Trust, Denmark Hill, London SE5 9RS, UK; abhishek.jha2@nhs.net; 4 ICU Department, Medical School, University Hospital of Alexandroupolis, Democritus University of Thrace, 68100 Alexandroupolis, Greece; vapapa@med.duth.gr

**Keywords:** dexmedetomidine, intensive care unit, critical care, delirium, sympatholytic, sleep in ICU, sedation

## Abstract

**Highlights:**

**What are the main findings?**

- The unique arousable sedation in combination with mild opioid-spare analgesic effects, has confirmed to effectively minimize duration of intubation and mechanical ventilation, ICU and hospital length of stay the total hospital stay cost.
- The anxiolytic and sympatholytic action have proved to sufficiently enhance sleep qualit, and has an important role on prevention and treatment of ICU delirium and post-ICU syndrome, especially among elderly patients.

**What is the implication of the main finding?**

- Increasingly evident advocate promising neuro-, renal-, and cardio-protective and anti-inflammatory effects of Dex, which are attributed to autophagy and apoptosis inhibition, sympatholytic, and ischemia/reperfusion (I/R) injury protective effect.

**Abstract:**

Background: Dexmedetomidine (Dex) is a well-known a2-adrenoceptor agonist with sedative, anxiolytic, sympatholytic, and analgesic effects that has been used principally as adjuvant sedation in the ICU. The enhanced clinical experience of Dex’s use and its physiological effects encourage its application beyond the initial indications. Aim: The purpose of this review is to summarize the current knowledge of Dex’s recently expanded applications in critically ill intensive care unit (ICU) adult patients. Methods: It is a narrative review that critically examines studies published since 2015 and referring to Dex’s use in ICU patients. Results: Despite the preliminary applications and the weak existing recommendation, the unique arousable sedation, in combination with mild opioid-spare analgesic effects, has been confirmed to effectively improve ICU outcomes. Moreover, the anxiolytic and sympatholytic actions have proved to sufficiently enhance sleep quality and prevent and treat ICU delirium and post-ICU syndrome, especially among elderly patients. Recently, increasing evidence advocates for promising neuro-, renal-, and cardio-protective and anti-inflammatory effects of Dex, which are attributed to autophagy and apoptosis inhibition and sympatholytic and ischemia/reperfusion (I/R) injury-protective effects. Conclusions: Beyond sedation, Dex seems to present promising neuroprotective, anti-inflammatory, and immunomodulating effects.

## 1. Introduction

Dexmedetomidine (Dex) is a highly selective α2-adrenoceptor agonist with sedative [1], antianxiety [2,3], sympatholytic, and mild analgesic opioid-sparing effects [4,5,6] without severe respiratory suppressive effects [7,8]. Unlike older commonly used sedative drugs, such as propofol and benzodiazepines, dexmedetomidine does not act at the gamma-aminobutyric acid (GABA) receptors. Compared with clonidine, another α2-agonist that has also been used for several decades, Dex has greater selectivity for α2-receptors (α2: α1 ratio of 1620:1 vs. 220:1), being a more potent sedative [9,10,11].

Its unique hypnotic action through the activation of central pre- and postsynaptic α2-receptors in the locus coeruleus induces a state of unconsciousness similar to natural sleep, while patients remain easily arousable and cooperative [7]. In addition, it has been shown to attenuate stress responses, minimizing the hemodynamic response to stressful events [3]. Its distinctive sedative and anxiolytic effects, with minimal influence on respiratory drive, support the consideration of Dex as a viable alternative sedative for critically ill patients in the intensive care unit (ICU).

In 1999, Dex was approved by the US Food and Drug Administration for short-term sedation of intubated and mechanically ventilated adults in the ICU and since 2008 in non-intubated patients during short-term surgical procedures, while since 2011 it has been approved in Europe for light to moderate ICU sedation [7,12]. Although the 2018 clinical practice guidelines for management of Pain, Agitation/Sedation, Delirium, Immobility and Sleep Disruption (PADIS) in adult ICU patients offered a conditional recommendation favoring the use of Dex over benzodiazepines and probably propofol in mechanically ventilated adults [13], in real clinical practice, midazolam is still frequently used, and propofol remains the main ICU sedative agent [14,15]. Moreover, the level of evidence for this recommendation was low since it was based on studies published between 2007 and 2015, and the quality of some of the included articles was questionable.

More recently, the Intensive Care Medicine Rapid Practice Guideline (ICM-RPG) issued a weak recommendation related to the use of Dex [16]. However, according to a recent systematic review and meta-analysis for PADIS treatment, Dex’s role and impact remain controversial due to the low quality of evidence [17]. The guideline task force provided moderate certainty recommendations for pharmacological management of agitation/sedation and sleep with Dex over propofol or benzodiazepines [18]. Moreover, Dex has gained popularity, appearing to be useful in multiple off-label applications in critically ill patients, broadening its clinical implementation.

The purpose of this narrative review is to summarize the current knowledge of dexmedetomidine’s more recently expanded applications in critically ill adult ICU patients, considering the physiological effects and highlighting properties that have not yet been implemented in common clinical practice.

## 2. Methods

This is a narrative review that critically examines recent literature indexed on PubMed since 2015 using the following key terms: “Dexmedetomidine”, “Intensive Care Unit”, “Critical care”, “Delirium”, “Adults” “Sleep in ICU”, “Sedation”, and “Sympatholytic”. The search was limited to peer-reviewed articles published in the last decade, in English, and involving clinical research or experimental studies, with a notable sample of adult critically ill patients, hospitalized in the ICU, and a few reviews and meta-analyses referring to the mechanism of action, pharmacodynamics, and pharmacokinetics.

**Figure 1:**
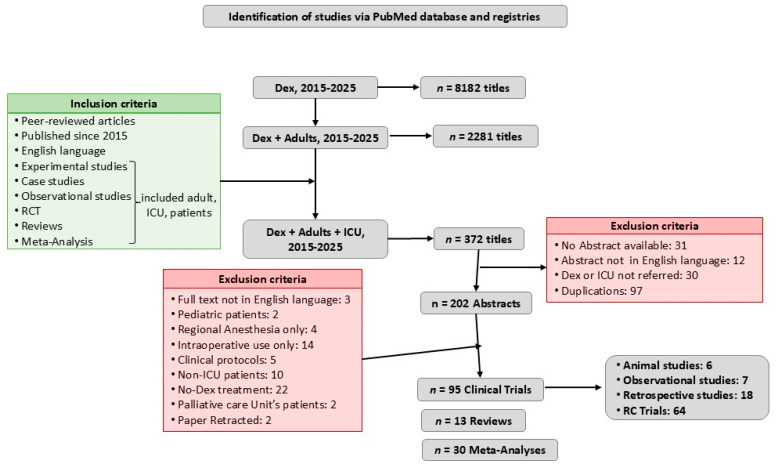
Flow diagram. This review included peer-reviewed articles in English, published since 2015, in PubMed. It contained all kinds of original clinical research (animal experimental studies, observational studies, retrospective studies, and randomized clinical trials (RCTs)) and a few relevant reviews and meta-analyses, principally referring to Dex’s mechanism of action, pharmacodynamics, and pharmacokinetics.

After screening the titles for possible relevance, and excluding duplicates, 202 papers were reviewed; 2 papers were retracted and 62 papers were excluded because the full text was not in English, included pediatric population, non-ICU, or palliative care unit population, or Dex was used only for regional or intraoperative anesthesia. The references of the articles were also reviewed for additional potentially relevant papers (Figure 1). The literature review was focused on efficacy of Dex as sedative, anxiolytic, analgetic, sympatholytic, neuroprotective, and anti-inflammatory agent and its impact on delirium, post-ICU care syndrome (PICS), and alcohol withdrawal syndrome (AWS) treatment.

## 3. Mechanism of Action

Dexmedetomidine is a dextrorotatory isomer of the racemic mixture medetomidine. It is an imidazole with the chemical name 4-[(1R)-1-(2,3-dimethylphenyl) ethyl]-3H-imidazole hydrochloride, with G-protein-coupled α2 receptor agonistic action. The α2 adrenergic receptors are widely distributed in the central nervous system (CNS), peripheral nervous system (PNS), autonomic ganglia, and other organ tissues, such as blood vessels, liver, kidney, pancreas, and platelets [19]. Different subtypes of α2 adrenergic receptors (α2A, α2B, and α2C) have different functions. Namely, α2A is considered to be the major presynaptic inhibitory feedback receptor, controlling the exocytosis of adrenergic neurons. Thus, α2 agonists have sedative, analgesic, and antiepileptic effects. In the brainstem, the locus coeruleus contains many α2-adrenoreceptors and plays a key role in wakefulness and regulation of nociceptive neurotransmission. Its agonistic action in the locus coeruleus inhibits norepinephrine release, resulting in depression of alertness and sympathetic activity that manifests sedation, analgesia, hypotension, and bradycardia [20].

The activated α2B receptors that are located mainly in peripheral vascular smooth muscle lead to transient hypertension [9], while those distributed in the spinal cord modify nitrogen monoxide analgesia by inhibiting noradrenergic action [21]. The α2C receptors, which are allocated in the hippocampus, basal ganglia, olfactory bulb system, and cerebral cortex, modulate complex memory and behavioral functions [8]. Dexmedetomidine is a highly selective activator of the α2A receptor, acting on the nucleolus of the nucleus, having sedative and hypnotic effects, while its action on the spinal cord can produce analgesic effects, and its action on the peripheral and CNS can play a role in inhibiting sympathetic excitation [9].

## 4. Pharmacokinetics and Administration

Dexmedetomidine in critically ill ICU patients is administered purely intravenously, with an action onset within 15 min and reaching peak concentration after 1 h of continuous infusion [19]. It has a half-life of 2 h and follows first-order linear kinetics. It is a highly protein-bound drug, binding almost 94% to plasma albumin and α1-glycoprotein. It is rapidly distributed since a half-life distribution of about 6 min in healthy volunteers has been reported [7]. The volume of distribution was found to be related to age, body weight [22], fat (free) mass [23], and serum albumin levels [24], being estimated at approximately 1.31–2.46 L/kg (90–194 L) in healthy people, although revealing high variability in ICU patients (109–223 L) [7,25].

It is mainly hepatically metabolized into inactive metabolites by glucuronidation and hydroxylation since less than 1% is excreted unchanged, with metabolites being excreted renally (95%) and intestinally (4%) [7]. Thus, patients with hepatic dysfunction may require lower doses due to its prolonged elimination [19,26,27]. Renal impairment does not affect its pharmacokinetics substantially, although it has been described as longer-lasting sedation in such patients, without significant differences in Dex plasma protein binding, volume of distribution, or clearance elimination [7,26]. In the elderly, sedation seems to be more pronounced, with hemodynamic side effects, such as severe hypotension and bradycardia, appearing more frequently, especially after loading doses of >0.7 μg/kg [7,28].

The recommended loading dose of Dex is 1 μg/kg over 10 min, followed by a continuous infusion of 0.2 to 0.7 μg/kg per hour, titrated to the desired level of sedation. A higher dose (1.5 μg/kg per hour) has been administered without clinically significant adverse effects. Body size [23], age [28], hepatic [27], cardiac and renal impairment, and hypoalbuminemia [24] have been found to pronounce pharmacological actions [7]. When Dex is administered to replace another sedative, a loading dose is generally not required since bolus and rapid dosage adjustment to achieve the desired level of sedation have been associated with more adverse drug events [19].

Dexmedetomidine can be administered for more than 24 h, safely in combination with anesthetics, sedatives, hypnotics, neuromuscular blockade agents, and opioids, in reduced doses. Similarly, co-administration with antihypertensive agents could also increase its hypotensive and bradycardic effects [29,30].

## 5. Effects of Dexmedetomidine

### 5.1. Sedation

Dexmedetomidine, in plasma concentrations between 0.2 and 0.3 ng/mL, induces the so-called “arousable or cooperative sedation” since it appears as an easy transition from sleep to wakefulness, keeping the patient calm, cooperative, and communicative when stimulated [31]. This sedative property, which simulates natural sleep, is caused by suppression of noradrenergic neuronal firing of locus coeruleus in the brain stem, leading to loss of wakefulness via activation of an endogenous sleep-promoting pathway. In contrast to opioids, benzodiazepines [32,33,34], or propofol [34,35], it diminishes respiratory depression, maintaining the hypercapnic arousal phenomenon even at higher doses, providing significant benefits in discomfort restraint during weaning procedures [7,31] (Table 1).

However, Lodenius et al. reported that, equivalent to propofol, dexmedetomidine reduces both hypoxic and hypercapnic regulation of breathing in young healthy volunteers at plasma concentrations of around 0.66 ng/mL [51], while it has been noticed that elderly patients are more vulnerable to respiratory depression [7]. Concentrations above 1.9 ng/mL could cause unarousable deep sedation [7], raising the frequency of cardiovascular side effects, minimizing the only Dex-based anesthesia strategy [31]. Although initially the US Food and Drug Administration approved Dex for use up to 24 h only, multiple studies showed a safety profile when continuous Dex sedation is used for up to 30 days in ICU patients [12].

Dexmedetomidine, was reported in two large European randomized controlled trials (RCTs) (PRODEX and MIDEX) to be non-inferior to propofol and midazolam, respectively, in maintaining light to moderate sedation in mechanically ventilated ICU patients [36]. The post hoc economic evaluation analysis of these RCTs demonstrated that sedation with Dex significantly shortened the time of extubation and length of mechanical ventilation, reducing ICU resource needs and costs in comparison with midazolam and propofol [12]. Similarly, Aggarwal et al. demonstrated that Dex was associated with lower costs when compared to propofol or midazolam, used for short-term sedation in ICU, due to minimized ICU length of stay (LOS) [40].

The Japanese open-label multicenter RCT (DESIRE study) examined the efficacy of Dex in 201 septic mechanically ventilated patients. They concluded that, although the use of Dex showed an 8% reduction in 28-day mortality compared with other sedatives, which could be clinically important, it did not demonstrate statistical significance. Moreover, even if the Dex group was reported to have more sufficient sedation, the delirium risk and the duration of mechanical ventilation were not reduced. The authors attributed Dex’s non-superiority in delirium prevention and mortality reduction to the limitations of RASS (Richmond Agitation Sedation Scale) and CAM-ICU (Confusion Assessment Method for ICU Patients) scores’ subjective assessment and the small sample size, respectively [29].

Furthermore, Brandão et al., in a large cohort of cardiac surgery patients, found that the use of Dex was associated with lower risk of 30-day mortality, shorter ICU LOS, and fewer postsurgical neurological lesions [37]. Nevertheless, this retrospective cohort study has some important limitations since the Dex group included younger patients, with lower disease severity, shorter operating time, and more often underwent off-pump surgery.

Zhou et al. revealed a notable reduction in weaning time, faster extubation, and lower delirium risk when midazolam was switched to Dex during a spontaneous breathing trial in mechanically ventilated ICU patients compared with propofol or continuation of midazolam [34]. However, many other smaller RCTs, with noteworthy limitations, compared Dex’s efficacy with midazolam [26,32] or propofol [35,38,43,44] and failed to show a clear benefit of Dex sedation according to different outcomes (Table 1).

The use of dexmedetomidine as the sole or primary sedative agent in critically ill patients was assessed in the SPICE III open-label multi-national RCT, where 4000 critically ill mechanically ventilated adults, from 74 ICUs in eight different countries, were randomized to receive either Dex or the usual care (propofol, midazolam, or other sedatives). Those who received early Dex required supplemental propofol, midazolam, or both more often than the usual care patients to achieve the desired level of sedation, assessed by RASS score, while no outcome or delirium risk benefit and more adverse events were reported [39]. The authors attributed their results to the lack of predetermined protocol for pain management, sedative titration, and delirium prevention.

Subsequently, in the secondary analysis of the SPICE III trial, the authors included only ICU mechanically ventilated patients aged older than 65 years old [41]. In this cluster analysis, the early use of Dex resulted in lower 90-day mortality compared to usual care in elderly patients, while, in younger patients, it appeared likely to increase mortality, especially in non-operative critically ill patients with high severity of illness. Smaller clinical trials verified that perioperative Dex infusion may improve ICU and hospital mortality rates in elderly cardiac surgical patients [28].

Another American multicenter double-blind trial (the MENDS-2 trial) that compared light sedation with Dex or propofol in 432 mechanically ventilated septic patients failed to show any differences in ventilator-free days, 28- and 90-day mortality, delirium, and post-ICU cognition [42]. The lack of pain and delirium prevention strategies was a notable limitation in this study.

Subsequently, an English large multicenter RCT (the A2B trial) revealed a notable reduction in mechanical ventilation duration among ICU patients being sedated with Dex in comparison with clonidine or propofol, although agitation and hemodynamical instability were described more often, and there was no difference in the final outcome [1]. The post hoc economic evaluation of the A2B trial revealed similar total cost and quality of life among the studied groups [45].

A Cochrane meta-analysis of seven studies and 1624 mechanically ventilated ICU patients compared long-term Dex sedation with traditional sedatives, such as midazolam, lorazepam, and propofol, and reported that Dex reduced mechanical ventilation duration by 22% and ICU LOS by 14% [46]. The lack of evidence for a beneficial effect on the risk of delirium and mortality was attributed to the high heterogeneity of the included studies [46]. Moreover, Lewis et al. reported reduced risk of delirium and shorter duration of mechanical ventilation and ICU LOS upon systematically reviewing 77 RCTs including 11,997 mechanically ventilated critically ill patients [49]. Previously, they reported reduced risk of intubation, delirium, and ICU LOS after reviewing 12 RCTs including 738 ICU patients treated with non-invasive ventilation (NIV) [47]. Similar results were presented by Wen et al. in their systematic review and meta-analysis, where they included 16 RCTs and 2035 ICU mechanically ventilated patients. They verified Dex’s benefit in regard to the risk of delirium, length of mechanical ventilation and ICU stay, and the total cost compared to midazolam [50] (Table 1).

### 5.2. Analgesic Effects

Analgesic effects of α2-agonists are thought to be mediated by α2-receptor binding in central and spinal cord α2-receptors. Pain transmission is suppressed by hyperpolarization of interneurons and reduction in the release of pronociceptive transmitters, such as substance P and glutamate [9]. Early studies, investigating the analgesic properties of dexmedetomidine, found that mild to deep sedation lacks analgesic efficacy compared to opioids. Therefore, it has been proposed to be combined with an opioid agent [7]. Moreover, according to Peng et al., this combination is more effective in minimizing opioid side effects [52]. However, recently accumulating evidence has demonstrated that dexmedetomidine may have an opioid-sparing analgesic effect on ischemic, acute postoperative [5,22,52], and refractory cancer pain [8] but limited efficacy in posttraumatic pain [6].

The mechanism of dexmedetomidine analgesia has not been fully clarified. Probably, analgesic effects of Dex may partly be owing to an altered perception and reduced anxiety, although an opioid-sparing effect is described [7]. The peripheral analgesic effect might be due to inhibiting the transmission of pain signals through Aδ and C fibers. Moreover, Dex depolarizes the blue plaque and the descending noradrenergic pathway of the spinal cord to the presynaptic membrane, inhibiting the release of substance P and other nociceptive peptides in the presynaptic membrane, thereby inhibiting the spinal cord via the transmission of angular noxious stimuli, which in turn terminates the signaling of pain. Furthermore, Dex seems to have a local analgesic effect through modulation of hyperalgesia by stimulating the α2 receptor [8].

### 5.3. Hemodynamic Effects

Dexmedetomidine produces a typical biphasic hemodynamic response, resulting in an initial transient hypertension phase combined with marked reflex bradycardia due to α2-receptor activation and peripheral vasoconstriction due to initial peak plasma concentration. After a few minutes, the vasoconstriction attenuates, resulting in vasodilatation. Together with presynaptic α2-adrenoreceptors inhibiting sympathetic release of catecholamines and increased vagal activity, this results in a hypotensive phase. An average decrease in mean arterial blood pressure of 13–27% has been observed and is maintained for a prolonged period after the initial dose. A sustained dose-dependent reduction in circulating plasma catecholamines has been related to these long-lasting sympatholytic effects [7].

Furthermore, dexmedetomidine inhibits the antidiuretic action of vasopressin, increasing diuresis, enhancing osmolal clearance, and preventing cortical blood flow [31]. Moreover, it has been reported that high Dex plasma concentrations are associated with significant increases in systemic and pulmonary vascular resistance, resulting in pulmonary and systemic hypertension, which can be a relevant contraindication in patients with previous history of advanced heart block or severe ventricular dysfunction. In such cases, the loading dose sizes or the rate of infusion could be decreased [7]. The dose-dependent bradycardia seen with Dex treatment is mediated primarily by a decrease in sympathetic tone and partly by baroreceptor reflex and enhanced vagal activity [31]. This sympatholytic activity reduces myocardial oxygen consumption by decreasing metabolism, thereby minimizing the incidence of myocardial ischemia and improving survival. However, it should be mentioned that hypotension resulting from dexmedetomidine can also be potentially pro-ischemic.

The main side effects of Dex, as a selective α2-adrenoceptor agonist, are bradycardia [1] and hypotension [29,33,39,47,50], especially among elderly patients [48] or those with volume depletion [19]. However, Chang et al. compared the effects of Dex and propofol on hemodynamics in surgical ICU patients after major surgery and reported no significantly different incidences of bradycardia, hypotension, and cardiac index reduction between the two groups [53]. The combination with medication with negative chronotropic effects, such as beta-blockers or amiodarone, could worsen bradycardia, resulting in left anterior fascicular block, first-degree AV block, or asystole [54].

Compared with propofol in septic shock patients, the risk of hemodynamic instability and bradycardia seems not to be significantly higher [55]. Previously, Morelli et al. demonstrated that, when propofol was switched to Dex in septic patients, maintaining the same depth of sedation, vasopressor requirements were reduced [56]. According to the A2B trial, patients in the Dex group appeared to have similar hypotension risk to those in the clonidine group but higher than those in propofol [1], while, in the SPICE III post hoc analysis, Dex, in critically ill patients with septic shock, appeared to be associated with lower vasopressor requirements to achieve the target mean arterial blood pressure compared to standard sedatives [57]. However, subsequently, the French ADRESS (a2 Agonist Dexmedetomidine for Refractory Septic Shock) trial failed to demonstrate a reduction in vasopressor resistance in patients with refractory septic shock, and the study was terminated early due to high mortality [58].

### 5.4. Delirium in ICU

Agitated delirium among critically ill patients is a common source of increased morbidity and mortality, impaired cognitive function, and extended mechanical ventilation and length of ICU stay since it increases the risk of self-extubation and removal of other essential medical devices [19,59,60]. However, it seems that Dex has both preventive and therapeutic effects in delirium, unlike other sedatives (Table 2).

#### 5.4.1. Delirium Prevention

It has been demonstrated that Dex-treated patients experience significantly less delirium in the ICU compared to patients treated with lorazepam [49], midazolam [49], or propofol [46,50,70,80] (Table 2). Many systematic reviews and meta-analyses support Dex’s possible perioperative delirium-preventive effect [80,81,82,83], especially among elderly patients [84], although they include a small number of heterogeneous RCTs, minimizing the strength of the evidence. This heterogeneity refers to the duration and time of Dex infusion (intra-, post-, or perioperative), the type of additional anesthesia (total intravenous or intravenous-inhaled combined), the severity and duration of the operation, age, and sample size.

A large number of clinical trials promote Dex’s possible delirium-preventive efficacy in ICU patients (Table 2). Particularly, MacLaren et al. reported that transitioning benzodiazepine sedation to Dex when patients qualify for daily awakenings may reduce delirium incidence and facilitate remembrance of ICU experiences without shortening the duration of mechanical ventilation [61]. Furthermore, Skrobik et al. evaluated Dex’s possible delirium preventive action through a two-center double-blind placebo-controlled RCT among ICU patients. They found that low-dose exclusively nocturnal Dex infusion reduced delirium incidence during ICU stay, although sleep quality remained unchanged, and length of mechanical ventilation and ICU and hospital LOS were similar between the two groups [62].

Moreover, a small Greek retrospective clinical trial compared Dex with standard of care in critically ill burn patients during weaning from mechanical ventilation. They found significantly lower rates of delirium and need for supplemental use of analgesic and antipsychotic agents in the Dex group, although the duration of mechanical ventilation was not reduced [44]. However, we have to mention that the patients in the Dex group were younger, suffering with a higher percentage of third-degree burn injury, evidence that could be a notable bias.

Conversely, after the Japanese DESIRE trial failed to reveal a clear delirium preventive benefit [29], Lee et al. also did not manage to prove any advantage of preventive infusion of Dex in postsurgical liver transplant patients, probably due to insufficient dose and duration and the unexpected low prevalence of delirium in both groups (<7.5%) [63]. In the DECADE study, Turan et al. suggested that the anti-inflammatory properties of a low dose of Dex may decrease postoperative delirium in cardiac surgery patients. However, the perioperative infusion of Dex did not decrease delirium onset in the treated group [64]. Similarly, He et al. could not demonstrate any delirium-preventive efficacy in postsurgical brain tumor patients, possibly because the incidence of delirium was not the primary endpoint of this study, the limited sample size, the presurgical affected neurological status, and the inadequate dose and duration of drug infusion [65].

Wang et al. compared the effect of sedation protocols with and without Dex on delirium risk and reported that the use of Dex could clinically slightly reduce the delirium risk, the ICU and hospital LOS, and the mechanical ventilation duration in ICU intubated patients [66]. They could not, however, find any benefit in mortality and the duration of delirium among the Dex-treated patients. They attributed these results to the small sample size and great heterogeneity of the 35 studies that were included in the meta-analysis [66].

The systematic review and meta-analysis by Heybati et al., which studied 41 trials including 3948 ICU mechanically ventilated patients, showed that Dex significantly reduced the duration of mechanical ventilation and the risk of ICU delirium in the cardiac surgical subgroup compared to propofol. Of note, the subgroup analysis also revealed that age might affect the incidence of hemodynamic side effects [48]. Another recent systematic review and meta-analysis, which included 16 controlled trials and 2035 ICU patients, compared Dex with midazolam and found a significantly shorter ICU LOS, duration of mechanical ventilation, and lower risk of delirium, showing a more obvious advantage in patients under 60 years old [50]. Of note, only 12 of the 16 RCTs (*n* = 1738 of 2035 patients) explored the incidence of delirium, while the included studies displayed statistical heterogeneity and small sample size.

#### 5.4.2. Delirium Treatment

Dexmedetomidine is also promoted as an effective agent for controlling agitation among critically ill patients [69] (Table 2). The effectiveness of Dex as an agitated delirium treatment in mechanically ventilated ICU patients was determined by the DahLIA multicenter double-blind placebo-control trial, where Dex, when added to standard care, was associated with more brief delirium resolution, earlier extubation, and shorter length of mechanical ventilation and ICU stay, with the benefits of reduced opioid requirements and minimal memory and cognitive function impairments [59] (Table 2). Although there was a difference in the primary and several congruent secondary outcomes, this study was underpowered to detect significantly different endpoints, such as ICU LOS, due to its small sample size and the notable heterogeneity in baseline characteristics, such as duration of ventilation before randomization.

Moreover, Lu et al. revealed that early midazolam switched to Dex in agitated intubated ICU patients improved stress response and hemodynamic stability during extubation, reducing duration of mechanical ventilation and incidence of delirium and ICU and hospital LOS [67]. Another single-center RCT compared Dex’s efficacy in delirium treatment compared to benzodiazepine in 18 ICU polytrauma patients and found lower serum neuron-specific enolase (NSE), S100 calcium binding protein B (S100B), and brain-derived neurotrophic factor (BDNF) levels in the Dex group, evidence that appears to be associated with effective delirium treatment [85].

Subsequently, in an Australian target trial, emulation analysis reported that early initiation of Dex in ICU patients with delirium was more effective in agitation resolution (94% vs. 72% 30-day delirium resolution in Dex group vs. controls, respectively), minimizing the length of mechanical ventilation and the need for tracheostomy. Subgroup analyses revealed increased agitation resolution within 30 days in patients older than 65, mainly postsurgical, non-septic, and nonventilated, who were treated with Dex earlier than 12 h after the onset of agitation [68].

Moreover, Liu et al. verified the efficacy of Dex in treating delirium by reviewing data from ten RCTs and five non-RCTs including 1017 critically ill patients suffering from delirium. Dex treatment significantly reduced the duration of delirium compared with placebo or other sedative drugs [69]. However, this meta-analysis includes only limited trials, characterized by notable heterogeneity regarding the study population characteristics (postsurgical, cardiac-surgery, or pathological patients), the intervention (drug dose and infusion duration), the control group, and the endpoints [69].

#### 5.4.3. Delirium Among Elderly Patients

Dexmedetomidine as both a preventive [70,71,73,74] and therapeutic agent for delirium has been examined more often among elderly ICU patients (Table 2). Djaiani et al. reported a significant prophylactic Dex benefit in frequency, time of onset, and duration of postoperative delirium in elderly patients after cardiac surgery compared to propofol [70]. However, patients who developed delirium in this single-center prospective RCT, which included 183 postsurgical cardiac patients aged >60 years, were older and had longer surgery and ICU LOS when compared with patients without delirium. The notable limitations of this study were the lack of blinding, the non-objective assessment of delirium using CAM and CAM-ICU scores, and the limited Dex infusion duration lasting less than 24 h, and then it was replaced by propofol. This practice may decrease Dex’s efficacy and probably explain the delayed delirium onset [70].

Moreover, Su et al. demonstrated that postoperatively prophylactic low-dose Dex (0.1 μg/kg/h) effectively prevented the occurrence of delirium and improved quality of sleep during the first 7 days in the ICU after non-cardiac surgery in elderly ICU patients aged >65 years (9% in Dex vs. 23% in placebo group; odds ratio 0.35, 95% CI 0.22–0.54; P < 0.0001) [71]. In addition, the DEXACET placebo-controlled RCT revealed that adequate postoperative analgesia and light sedation with Dex or propofol prevented delirium incidence among elderly cardiac surgical patients [73]. It has also been reported that postoperative Dex infusion via patient-controlled intravenous analgesia significantly minimized delirium prevalence, although it did not modify length of ICU and hospital LOS and mortality, among elderly patients undergoing thoracoabdominal tumor surgery [74].

Nevertheless, Deiner et al. reported that the intraoperative administration of Dex did not prevent postoperative delirium among elderly patients undergoing major elective non-cardiac surgery, possibly due to the short-acting nature of the drug and loss of salutary effects after discontinuation of the infusion [72]. The DIRECT trial was also underpowered to detect any preventive effect of Dex among elderly patients after undergoing cardiac surgery, probably due to its small sample size and the subjectivity of delirium diagnosis and assessment [43]. Similarly, Huet et al. failed to prove any delirium preventive benefit of Dex’s overnight infusion in older cardiac surgical patients, likely due to short-lasting drug action [75].

The effectiveness of Dex on delirium in elderly surgical patients was studied through a systematic review and meta-analysis of 21 trials including 6328 participants, where they concluded that Dex obviously decreased delirium occurrence in non-cardiac but not in cardiac elderly surgical patients [77]. Of note, only five of the twenty-one included studies assessed delirium in 1217 cardiac surgery patients, and the remaining sixteen studies included 5111 non-cardiac surgery patients. In addition, the authors remarked that there was moderate heterogeneity among all the RCTs regarding the time (intra-, peri-, or postoperative), the dose and the duration of Dex’s infusion, and the control group (placebo, propofol, or other sedative agent) [77].

Previously, Pereira et al. compared the delirium-preventive effect of Dex sedation with propofol through a systematic review of seven studies (six RCTs and two retrospective cohorts) that included 1249 elderly ICU patients and reported a lower delirium risk without raising adverse event frequency or outcome benefit [76]. It is important to note that this meta-analysis included notably heterogeneous studies regarding the study population (cardiac, non-cardiac surgical patients, or mixed medical and surgical ICU patients), the tool for delirium diagnosis, and the sedative protocol [76]. Recently, the protocol of the ALPHA 2 PRESENT Norwegian multicenter RCT was published, where the authors intended to evaluate the efficacy of Dex compared to clonidine or placebo in delirium prevention among elderly cardiac surgical patients [86]. Similarly, the protocol of the French EXACTUM placebo-controlled multicenter RCT has been announced, where Dex’s delirium-preventive effectiveness will also be assessed in cardiac postsurgical ICU patients older than 65 years [87].

#### 5.4.4. Comparative Efficacy

Compared to clonidine, Dex has eight times more affinity for α2-adrenoreceptors, with more effective sedative and analgesic properties [19], while, in contrast to haloperidol, it has been described to be associated with fewer electrocardiographic changes in QT and QTc interval [88] (Table 2).

Carrasco et al. evaluated the clinical efficacy of Dex in non-intubated critically ill patients with agitated delirium refractory to haloperidol and reported better effectiveness, safety, and cost–benefit in cases where haloperidol failed to control delirium and was replaced by Dex [78]. Moreover, Shokri et al. evaluated the prophylactic efficacy of Dex and clonidine on rate and duration of delirium in older cardiac surgery patients. In this RCT, the authors found that postoperative Dex infusion was associated with lower risk and duration of delirium, shorter mechanical ventilation duration and ICU LOS, lower mortality rates, and lower morphine consumption than the clonidine group [79]. The major limitation of this study is that it is a single-center study with a small sample size, including possible bias regarding institutional standards of care. Furthermore, recently, the Japanese DEX-HD RCT was announced to compare the efficacy of Dex versus haloperidol in the treatment of nocturnal hyperactive delirium in non-intubated patients in high-dependency units [89].

Dexmedetomidine, as a sympatholytic a2-adrenergic agonist, is proposed to reduce stress response compared to commonly used sedatives in critically ill patients. Moore et al. measured plasma levels of stress response biomarkers in critically ill ventilated patients and showed that early sedation with Dex could not significantly change the physiological and serum parameters associated with the stress response as with midazolam or propofol [3]. On the other hand, Dex seems to decrease plasma stress hormones in cardiac surgery patients, when infused intraoperatively, compared with a placebo [2]. Zi et al. reported that perioperative Dex was related to significantly lower anxiety levels, after off-pump coronary artery bypass graft, compared with propofol [90].

#### 5.4.5. Sleep Quality Improvement in ICU

Critically ill patients suffer from seriously disrupted sleep [91,92] and markedly segmented circadian rhythm [93]. Poor sleep quality has been related to delirium, long-lasting cognitive impairment, prolonged ICU and hospital stay, and increased morbidity and mortality [94]. Dexmedetomidine has been reported to more closely resemble natural non-REM sleep compared to GABA agonists, facilitating patient–caregiver interaction [91] (Table 3).

Wu et al. reported that prophylactic low-dose Dex infusion in non-intubated non-cardiac postsurgical elderly ICU patients improved and prolonged nocturnal sleep compared with a placebo [95]. Zhang et al. also randomized patients older than 65 years old, following non-cardiac surgery, to receive patient-controlled intravenous analgesia supplemented with either Dex or placebo [98]. In line with previous results [91,95,96], the authors showed that the Dex group had improved sleep structure and prolonged total sleep time [98]. In the MINDSS study, Qu et al. reported that a single bolus of Dex administered at nighttime could prevent delirium by promoting sleep. However, no difference was found regarding sleep quality as Dex is a short-acting drug and a single dose may not be sufficient to promote improved sleep quality [97].

Moreover, Dex treatment seems to increase total sleep duration and improve sleep efficiency in non-intubated ICU patients [94,95,98,99] and in mechanically ventilated patients [91,96] compared to a placebo or no sedation.

A recent systematic review of 29 trials including 5610 non-cardiac postoperative ICU patients explored the effects of perioperative Dex on postoperative sleep quality [100]. They concluded that Dex may improve the early postoperative quality pattern, although the evidence was of low quality.

#### 5.4.6. Post-Intensive Care Syndrome

The term post-intensive care syndrome (PICS) refers to the current or worsening impairment of mental health, cognition, psychological, and physical vigor following critical illness [111]. Improvements in ICU patients’ short-term outcomes revealed the burden of survivors’ long-term quality-of-life issues. Perioperative treatment with dexmedetomidine has been associated with better postoperative analgesia and decreased opioid utilization [29], better quality of sleep [91,94], and minimized inflammatory response [112,113] —all of which could worsen post-ICU psychological and cognitive impairment [111]. Dong et al. reported significantly reduced rates of PICS and improved 6-month mortality in cardiac postsurgical patients treated with prophylactic nocturnal Dex administration [101] (Table 3).

#### 5.4.7. Alcohol Withdrawal Syndrome

Alcohol withdrawal syndrome (AWS) occurs after a period of absolute or relative abstinence from alcohol, where blood alcohol levels decrease significantly in habituated individuals [114]. Diagnosis of AWS in ICU patients is associated with the need for excessive doses of sedatives, longer mechanical ventilation, ICU and hospital length of stay, and inflated healthcare costs [114]. Approximately 10–30% of ICU patients experience AWS, typically beginning within 6 to 24 h after alcohol cessation, peaking 36 h post-abstinence. The first-line treatment includes γ-aminobutyric acid agonists, such as benzodiazepines, for controlling persistent autonomic hyperactivity and preventing complications like seizures or delirium tremens [107,115]. The adjunctive agents evaluated include α2-agonists.

Intravenous dexmedetomidine has been reported to reduce neuronal loss in the locus coeruleus and catecholamine neuron degeneration, improving AWS symptoms [103]. Because of its anxiolytic and sedating effects, it can reduce benzodiazepine dosages, as shown in two retrospective cohort studies [102,103] (Table 3). In the first study, the 12-h change in benzodiazepine requirements differed significantly in 20 AWS patients treated with Dex compared to 22 treated with benzodiazepines alone [102]. The second cohort included 77 patients admitted to the medical ICU with severe AWS, showing that Dex improved Clinical Institute Withdrawal Assessment (CIWA) scores and benzodiazepine requirements, although it prolonged ICU length of stay [103].

In line with the above results, the systematic review of Woods et al., which included four studies with 55 AWS ICU patients, found that adjuvant Dex treatment added to benzodiazepine-based therapy reduced delirium risk and improved AWS control more effectively than benzodiazepine therapy alone [108]. Similarly, a second review by Wong, which studied thirteen articles (eight case reports/series and five RCTs), also reported the possible Dex adjuvant’s role in benzodiazepine-based therapy in AWS ICU patients [107]. It is important to note that both these reviews included heterogenic, small, and low-quality studies.

However, combining Dex with benzodiazepines or phenobarbital seems to prolong mechanical ventilation, ICU stay, and duration of delirium [104,106] without expected efficacy; studies have failed to demonstrate notable changes in CIWA score, intubation risk, or seizure control [104,105]. Dex possibly suppressed AWS signs and symptoms without treating the underlying withdrawal physiology as it has no GABA modulation effects, altering the kinetics of withdrawal in a manner that prolonged its duration. Additionally, Dex’s infusion requires ICU level of care due to potential cardiovascular complications. Thus, those patients who received prolonged Dex infusion probably remained in ICU because of the drug monitoring requirements [104].

Similarly to the above, Polintan et al., in a review of 12 studies, reported no significant benefit of adjunctive Dex added to benzodiazepine over benzodiazepine monotherapy regarding ICU and AWS control [109]. More recently, Fiore et al. also evaluated Dex as an adjunctive therapy for AWS and found that Dex failed to reduce intubation rates in patients with AWS, while it increased bradycardia risk [110]. Both of these meta-analyses included small low-quality heterogeneous studies.

Dexmedetomidine has been reported to have potential neuroprotective effects since it seems to dose-dependently decrease intracranial pressure, cerebral blood flow, and cerebral metabolic rate [21,84,116]. The underlying possible pathophysiologic mechanism for these neuroprotective effects is complicated and unclear. Sympatholytic effects of dexmedetomidine could minimize hypoxic brain damage, which is worsened through catecholamine release in nerve synapses since the latter increases neuronal sensitivity to glutamate during neuronal ischemia and promotes oxidative stress, worsening the existing nerve tissue impairment [8]. It also seems to reduce neuronal damage by inhibiting neurotransmitter release, which improves ischemic perfusion and metabolic disorders [117] (Table 4).

Animal studies presented reductions in neuronal cell death in the cortex and the hippocampus and a prevention effect on axonal damage and synaptic degeneration in the cortex while improving functional recovery after traumatic brain injury (TBI) [118]. A remarked limitation of this in vivo study is that the mice treated with Dex were obviously hypothermic. Thus, the demonstrated neuroprotective effects on TBI may not only be due to Dex’s activation of the signal transduction cascade but also the hypothermia induced by Dex [118]. Moreover, Feng et al. reported a significant alleviation of neurological deficits and brain edema in a TBI animal model, which was attributed to Dex-mediated inhibition of autophagy and neuroinflammation, ameliorating neuronal apoptosis related to the ROS/Nrf2 pathway [119]. Similarly, Li et al. demonstrated that Dex minimizes post-TBI inflammatory response by restriction of NF-κB activation through the Nrf2 signaling pathway [85], while Huang et al. imputed its anti-inflammatory and neuroprotective effects to its possible action on the TLR4/NF-κB pathway [120].

It has also been reported that dexmedetomidine presents neuroprotection in hypoxic injury through inhibition of NF-κB/COX-2 pathway activation [126]. Moreover, a protective effect on ischemia/reperfusion (I/R) injury has been described, preventing neuronal cells’ apoptosis through an intrinsic Bax–mitochondria–cytochrome c-caspase protease pathway [118].

Furthermore, retrospective studies based on the MIMIC-IV database found that adjuvant sedation with Dex was correlated with better outcomes among patients with aneurysmal subarachnoid hemorrhage (aSAH) [85,124], ischemic stroke [122,123], and TBI [116,121]. Possibly, Dex presented a protective effect in SAH patients either by anti-inflammatory or inflammation-modulatory properties, regulating three specific hub genes (MyD88, AR, and AREG), which have been identified as potential mediators of its protective effects [85], or by improving hemodynamics and cerebral perfusion, minimizing delayed cerebral ischemia [124]. Of note, Dex infusion did not improve the prognosis of patients with a low Charlson score ischemic stroke but instead provided greater clinical benefits for patients with multiple comorbidities, suggesting that, except from its neuroprotective effects, Dex may reveal benefits for other chronic diseases [123].

In line with this evidence, Lu et al. assessed the neuroprotective efficacy of Dex in TBI patients through internal jugular vein catheterization and evaluation of SjvO_2_, CEO_2,_ and serum interleukin 6 (IL-6) and −1*β* (IL-1*β*) levels [125]. In the Dex group, they found significantly enhanced cerebral oxygen metabolism and reduced inflammatory biomarkers [125]. Moreover, a meta-analysis, which included 19 RCTs and 879 patients, amplified Dex’s possible neuroprotection effect on ischemic brain injury, attributed to hemodynamic stability, control of inflammatory and neuroendocrine response, and maintenance of intracranial homeostasis [21].

#### 5.4.8. Anti-Inflammatory Effects

Dexmedetomidine is proposed to have cardio- and renoprotective and anti-inflammatory properties [7] (Table 5).

#### 5.4.9. Cardio-Protective Properties

Dexmedetomidine as a sedative adjuvant has been proposed to modulate the inflammatory response after coronary artery bypass surgery (Table 5). Bolow et al. reported a significant reduction in postsurgical inflammatory biomarkers (IL-1, -6, TNF-α, INF-γ, and C-reactive protein) when Dex was used as an adjuvant sedative in cardiopulmonary bypass patients compared with non-Dex sedation. These findings indicate that Dex possibly modifies the inflammatory response in such cardiac surgery [112]. Moreover, Liu et al. compared the effects of Dex and propofol on sublingual microcirculation in patients after cardiac surgery and showed an earlier and greater improvement in the Dex group compared with propofol [127]. The possible mechanisms they proposed include inflammatory attenuation, decrease in leukocyte-endothelial interactions, mild hypocoagulation, and reduction in capillary perfusion deficits [127].

Furthermore, another small prospective RCT indicated a notable anti-inflammatory and myocardial protective effect when Dex was administered before anesthesia induction in patients during cardiac valve replacement [128]. In addition, the review of Zhang et al. evaluated the effect of Dex on myocardial I/R injury in patients undergoing cardiopulmonary bypass [129]. They included 17 retrospective studies, which involved 843 patients, and reported that the addition of Dex can significantly reduce the serum levels of CK-MB and troponin after cardiopulmonary bypass, suggesting its possible myocardial tissue protective effect [129].

In addition, further studies reveal that dexmedetomidine exerts dose-dependent renoprotective effects following surgery or during sepsis, likely attributed to anti-inflammatory, cytoprotective, and sympatholytic effects (Table 5). Cakir et al. demonstrated, in a kidney I/R experimental model, that Dex reduced I/R injury (lower levels of malondialdehyde, catalase, and glutathione), antioxidant enzyme activity, kidney dysfunction (lower levels of creatinine (Cr) and blood urea nitrogen (BUN)), and histologic injury score [130]. Of note, according to their results, applying 100 μg/kg of Dex instead of 10 μg/kg seems to be more effective in terms of healing kidney I/R damage [130]. A second animal study also investigated the possible protective effects of Dex in rat sepsis models [131]. They found that, when rats were treated with Dex, Acute Kidney Injury (AKI), induced by sepsis, was decreased significantly. In addition, animal model exposure to an a2-adrenergic receptor antagonist (yohimbine) eliminated this reduction [131].

The results of these experimental studies have been verified by two RCTs [113,132]. The first one, a small prospective randomized controlled placebo trial, showed that pretreatment with Dex in patients undergoing cardiac valve replacement under cardiopulmonary bypass attenuated renal injury and decreased the incidence of AKI [113]. This possibly renoprotective effect was associated with increased superoxidase activity [113]. The second RCT aimed to compare Dex with propofol and the incidence of AKI in septic mechanically ventilated patients [132]. The authors reported a significantly lower level of renal injury (Cr and BUN) and inflammatory biomarkers (TNF-α, IL-1, CD4+, and CD8+ T lymphocytes) in the Dex group, indicating a significant decrease in AKI incidence and renal replacement demands due to the possible anti-inflammatory and immunoregulatory action of Dex [132].

Dexmedetomidine has been shown to confer possible renoprotection by stabilizing the sympathetic system, exerting anti-inflammatory effects, and attenuating I/R injury. Liu et al. verified these findings after systematically reviewing 10 trials including 1575 postsurgical cardiac patients, reporting a significant reduction in postoperative AKI incidence [133]. However, this meta-analysis is based on low-quality evidence since it examined a limited number of trials with great heterogeneity regarding the studied population (coronary artery bypass or valve replacement; on- or off-pump), the intervention (Dex vs. placebo, or Dex vs. different sedatives), and adjuvant sedatives and analgesics [133]. A second meta-analysis by Zhao et al. that studied 15 trials, enrolling 2907 cardiac surgery patients, verified that perioperative Dex reduced the incidence of postoperative AKI [134]. Of note, the subgroup analyses revealed similar trends regardless of age, comorbidities, and the dose and duration of Dex infusion [134].

Finally, a recent high-level-of-evidence meta-analysis of 25 trials including 3997 postoperative patients revealed a significant reduction in AKI occurrence among individuals who were administered Dex in contrast to the control group [135]. The following subgroup analyses demonstrated that Dex did not have a statistically significant influence on subjects who underwent non-cardiac operations. This could be linked to the relatively lower incidence of AKI in non-cardiac surgery patients compared to cardiac patients, while the sample-size of the meta-analysis for non-cardiac patients was small [135].

#### 5.4.10. Dexmedetomidine in Sepsis

Dexmedetomidine has been shown to minimize inflammatory response during sepsis in experimental models of sepsis [131]. However, data from clinical trials are limited (Table 5). An open-label pilot RCT by Iten et al., which compared Dex-based sedation with propofol- or midazolam-based sedation, failed to show any significant difference in septic encephalopathy biomarkers (S100-β) in critically ill patients with sepsis requiring mechanical ventilation [137]. These results were attributed to the small sample size (34 patients in the Dex group and 36 in the control group) and the increased dose of added propofol or midazolam in the Dex group in order to achieve sedation effectively [137]. Similarly, Patidar et al. revealed that Dex improves regional cerebral oxygen saturation in septic patients compared to propofol (utilizing Near-Infrared Spectroscopy monitor-NIRS), although no differences in delirium occurrence and duration were detected [138].

Moreover, the DecatSepsis trial proved to be underpowered to detect any significant reduction in mortality or norepinephrine dose in septic patients treated with Dex [136]. In addition, the ADRESS multicenter RCT, which was designed to evaluate Dex’s efficacy on the vasopressor response in patients with refractory septic shock, also failed to reveal any benefit and was terminated prematurely due to significantly higher mortality in the Dex arm [58]. Of note, sepsis is a complicated and heterogenic syndrome caused by a dysregulated host response to infection [142]. It is at least simplistic to try to correlate sepsis’ outcome with a single intervention, like Dex infusion.

Furthermore, Chen at al. examined the literature in order to determine Dex’s influence on mechanical ventilation duration among septic patients [139]. They reviewed only four studies and enrolled 349 patients, but they reported a significant increase in ventilator-free days and better 28-day survival in those patients treated with Dex [139]. In line with the above results, a second meta-analysis by Wang et al. failed to reveal any benefit of Dex use in septic patients compared with common sedative agents regarding mortality, ICU LOS, delirium incidence, and duration [140].

Subsequently, another meta-analysis by Zhang et al. included 19 RCTs that enrolled 1929 ICU septic patients treated either with Dex or other sedatives [141]. They reported significantly better survival in the Dex group compared with midazolam but not with propofol, and there was a notable elimination of inflammatory response. In line with the previous results, there were no differences in ICU LOS, mechanical ventilation duration, incidence of delirium, and rate of organ dysfunction [141].

## 6. Recommendations for Practice and/or Further Research

Dexmedetomidine seems to be an attractive alternative anesthetic for critically ill patients, with a variety of clinical indications. However, certain extended applications of Dex, such as neuroprotection or immunomodulation, require further evaluation.

## 7. Conclusions

In conclusion, compared with the current pharmacological management strategies, dexmedetomidine appears to have some advantages considering the pharmacokinetic profile of the drug. It has high specificity and selectivity for α2-adrenoreceptors and blocks norepinephrine, disrupting neurotransmitter pathways. It presents sedative effects that are comparable to GABA agonists, without respiratory depression, promoting a more physiological sleep–wake cycle, thereby prolonging sleep and improving its quality. As a result, Dex seems to eliminate the risk of delirium and post-ICU syndrome, especially among elderly and cardiac surgery ICU patients. Moreover, the latest evidence suggests a variety of extended indications for its use, such as neuroprotection as well as anti-inflammatory and immunomodulatory properties. These clinical applications require further evaluation and careful patient selection.

## Figures and Tables

**Table 1:** Dexmedetomidine as sedative in ICU patients.

	Study Design	Study Population	Intervention	Comparison	Main Outcomes	Secondary Outcomes	Adverse Events	Study Limitations
Turunen H, et al., 2015 [12]	Post hoc analysis of MIDEX and PRODEX study (Jakob SM, et al. JAMA 2012;307:1151–60) [36]	990 ICU adult patients requiring prolonged MV	493 Dex group 250 midazolam (MIDEX) 247 propofol (PRODEX)	Dex vs. propofol or midazolam	Reduce total ICU resource utilization and respective costs	- Shortening the extubation time and - MV duration, especially in comparison with midazolam	Not referred	- Great heterogeneity of study population. - ICU patients with severe neurological disorders were excluded.
Gupta S, et al., 2015 [32]	RCT	40 ICU MV patients	0.2–0.7 mcg/kg/h Dex vs. 0.04–0.2 mg/kg/h midazolam	Dex vs. midazolam	Dex significantly minimized time to extubation	Easy arousability, Lack of respiratory depression	Not referred	A small single-center study
Cheng H, et al., 2016 [28]	Single-center Retrospective clinical trial	505 ICU CAP patients, aged > 65 years	0.24–0.6 μgr/kg/h Dex	Dex vs. other sedatives	Dex decreased in-hospital (0.90 vs. 2.83%) and operative mortality (1.35 vs. 3.18%)	Dex reduced risk of stroke (0.9 vs. 1.77%), delirium (7.21% vs. 10.95%)	Not referred	A single-center non-randomized cohort study, with a relatively small sample size
Brandão PGM, et al., 2016 [37]	Single-center Retrospective clinical trial	1302 ICU CAP patients	0.5 μgr/kg/h Dex	Dex vs. other sedatives	Dex was associated with lower 30-day mortality (3.4 vs. 9.7%)	Shorter ICU LOS (3.1 vs. 7.3%) Decreased incidence of neurological problems	Not referred	- A single-center retrospective study - Dex patients were younger and underwent more often off-pump surgery - Time of CPB was shorter in DEX group
Kawazoe Y, et al., 2017 [29]	Multicenter RCT (8 ICUs in Japan) (DESIRE trial)	201 septic MV patients	Sedation with Dex vs. without Dex		Same 28-day mortality (22.8 vs. 30.8%) and Ventilator-free days (20 vs. 18)	Dex group had higher rate of well-controlled sedation but no reduction in delirium	Higher rates of bradycardia in Dex group	- An open-label study - The end points were assessed by physicians at discharge - RASS and CAM-ICU scores were assessed by “not blinded” nurses - Only short-term outcomes were analyzed
Elgebaly AS, et al. Ann Card Anae [38]	Single-center RCT	50 ICU CAP patients, aged 18–55 years	0.8 μgr/kg/h Dex vs. 1.5 mg/kg/h propofol	Dex vs. propofol	No difference between groups in extubation time, analgesic requirements, or ICU and hospital LOS	Same hemodynamic stability, Increased financial costs in Dex group	Not referred	- Small sample size - Different cardiovascular operations (CABG or valve/CABG) with different possibility for postsurgical respiratory failure - Many subjects required more than one sedative agent, increasing MV duration
Shehabi Y, et al., 2019 [39]	Multicenter RCT (SPICE III trial)	4000 patients	Dex as sole or primary sedative or usual care	Dex vs. other sedatives	Same 90-day mortality,	Dex group required supplemental sedatives	Higher rates of bradycardia and hypotension in Dex group	- Unblinded RCT - Patients that required deep sedation were not excluded - No sedation daily interruption - No sedation managing protocol - No delirium prevention protocol
Wang L, et al., 2020 [26]	Single-center RCT	40 oral and maxillofacial postsurgical ICU, intubated patients	0.1 μgr/kg/h Dex vs. 0.2 mg/kg/h midazolam	Dex vs. midazolam	BPS and Ramsay scores were higher in midazolam group. Shorter time of extubation in Dex group.	Lower incidence of delirium and respiratory depression in Dex group.	Higher rates of bradycardia in Dex group	A small single-center study
Aggarwal J, et al., 2020 [40]	A single-center cost analysis study	Short term sedation in ICU, MV patients	Dex vs. other sedatives		Dex reduced costs/person Dex reduced ICU LOS		Not referred	- A single-center open label trial - The cost analysis model was suitable for US national healthcare system
Winings NA, et al.,2021 [35]	Single-center RCT	57 trauma MV ICU patients	Dex vs. propofol		No significant difference in MV duration (125 vs. 130 h) Dex achieved better target sedation	No difference in mortality, ICU and hospital LOS, or incidence of delirium.	Similar incidences of bradycardia and hypotension	- Single-center open label study - Patients in Dex group spent more time in target sedation
Shehabi Y, et al., 2021 [41]	Secondary analysis of SPICE III trial	1825 ICU patients, MV more than 24 h, aged > 65 years	Dex vs. standard sedation	Dex vs. other sedatives	Reduced 90-day mortality in patients >65 years old	High probability to increase 90-day mortality in younger non-operative patients	No difference	- A secondary post hoc analysis - Lack of blinding - Absence of a strictly protocolized strategy fer sedation or delirium management
Hughes CH, et al., 2021 [42]	Multicenter RCT (MENDS 2 trial)	432 ICU MV septic patients	Dex vs. propofol		No difference in mortality, Delirium risk, or ventilator-free days		Not referred	Lack of pain and delirium prevention strategy
Zhou Y, et al., 2022 [34]	Single-Center RCT	252 ICU, MV patients, long lasting weaning	0.2–0.7μ g/kg/h Dex plus midazolam vs. plus propofol vs. midazolam alone during SBT		When midazolam was switched to Dex, faster extubation	Less weaning time, Lower delirium,	No difference in adverse events rate	- Unblinded single-center trial - The use of midazolam was not in accordance with the PAD 2013 and PADIS 2018 guidelines
Chitnis S, et al., 2022 [43]	Single-Center RCT (DIRECT study)	70 postsurgical cardiac patients, older than 75 years old	Dex vs. propofol		Decrease in MCAS score in Dex group	No difference in delirium rate, time to extubation, length of hospital stay	No difference in adverse events rate	- A small open-label single-center study, - Only postsurgical cardiac patients.
Stangaciu B, et al., 2022 [44]	Single-Center Retrospective clinical trial	56 MV, burn ICU patients, during weaning	Dex 1 mcg/kg bolus and 0.4–0.1 mcg/kg/h iv vs. 0.08 mg/kg/h midazolam or 15–30 mcg/kg/min propofol	Dex vs. midazolam or propofol	No significantly shorter duration of MV (9.3 vs. 7.5 days), Lower delirium rate (38.4 vs. 53.3%) (assessed with CAM-ICU score),	Less need for supplemental analgesia (23.1 vs. 53.3%) (assessed with RASS score), Less need for antipsychotic agents (15.4 vs. 53.3%)	- Bradycardia - Abdominal distention	- A small retrospective single-center study. - Patients with Dex sedation were younger and had higher percentage of 3rd-degree burn injuries.
Walsh TS, et al., 2025 [1]	Multicenter Open-label RCT (41 ICUs in UK) (A2B trial)	1404 adult, MV, ICU patients	Propofol + opioids vs. Dex or clonidine + opioid	Dex or clonidine vs. propofol	Reduced MV duration (Dex: 136 h, clonidine: 146 h and propofol: 162 h)	More agitation in Dex group and clonidine vs. propofol, Similar mortality	Higher rate of bradycardia in Dex and clonidine group	
Morris S, et al., 2025 [45]	Multicenter Open-label RCT (41 ICUs in UK) (economic evaluation of A2B trial)	1404 adult, MV, ICU patients	Propofol + opioids vs. Dex or clonidine + opioid	Dex or clonidine vs. propofol	Similar costs and QALYs		Not referred	A post hoc economic evaluation of previous RCTs
Chen K, et al., 2015 [46]	Systematic Review of 7 studies	1624 adult patients requiring prolonged MV	Dex vs. propofol, or midazolam, or lorazepam		Reduced duration of MV by 22%	Reduced length of ICU stay by 14%		
Zhou WJ, et al., 2021 [33]	Systematic Review of 8 studies	1379 patients	Dex vs. midazolam		Dex reduced LOS in ICU, time to extubation, delirium	No difference in hypotension incidence and mortality	Higher incidence of bradycardia with Dex	
Lewis K, et al., 2021 [47]	Systematic Review of 12 RCTs	738 NIV patients	Dex vs. other sedatives		Reduced the risk of intubation Reduced ICU LOS	Reduced risk of delirium	- Bradycardia - Hypotension	-
Heybati K, et al., 2022 [48]	Systematic Review of 41 studies	3948 MV ICU patients	Dex vs. propofol		No difference in ICU LOS	Reduced MV duration and delirium risk **only** among cardiac surgical patients	- Bradycardia, - Hypotension, **especially** among elderly	
Lewis K, et al., 2022 [49]	Systematic Review of 77 RCTs	11,997 patients MV ICU patients	Dex vs. other sedatives		Reduced MV duration Reduced ICU LOS	Reduced delirium risk	- Bradycardia, - Hypotension	-
Wen J, et al., 2023 [50]	Systemic Review of 16 RCTs	2035 patients	Dex vs. midazolam		- Shorter LOS ICU - Lower risk of delirium - Shorter duration of MV	- No difference in mortality and - length of hospital stay	- Bradycardia, - No difference in hypotension	

**Abbreviations:** BPS: Behavioral Pain Scale, CABG: coronary artery bypass graft, CAP: cardiopulmonary bypass, Dex: dexmedetomidine, h: h., ICU: intensive care unit, LOS: length of stay, MCAS score: Minnesota Cognitive Acuity Screen score, MV: mechanical ventilation, PAD: Pain, Anxiety and Delirium, PADIS: Pain, Anxiety, Agitation/Sedation, Delirium, Immobility and Sleep Disruption, QALYs: quality-adjusted life years, RCT: randomized controlled trial, SBT: spontaneous breathing trial, UK: United Kingdom.

**Table 2:** Indications for dexmedetomidine in ICU delirium.

	Study Design	Study Population	Comparison	Assessment of Delirium	Main Outcomes	Secondary Outcomes	Study Limitations
*2.1 Delirium Prevention*							
MacLaren R, et al., 2015 [61]	Single-center double-blind RCT	11 ICU MV patients in weaning time received Dex vs. 12 midazolam	0.61 μg/kg/h Dex vs. 3.7 mg/h midazolam	- CAM-ICU	Dex reduced delirium incidences	- Rates of anxiety and depression were similar. - Higher risk of hypotension in Dex group	- A small single-center trial
Kawazoe Y, et al., 2017 [29]	Open-label, multicenter RCT (8 ICUs in Japan) (DESIRE trial)	201 ICU septic MV patients	Sedation with Dex vs. without Dex	- CAM-ICU	Same 28-day mortality (22.8 vs. 30.8%) and Ventilator-free days (20 vs. 18)	- Dex group had higher rate of well-controlled sedation but - no reduction in delirium	- An open-label study - The end points were assessed by physicians at discharge - RASS and CAM-ICU scores were assessed by “not blinded” nurses - Only short-term outcomes were analyzed
Skrobik Y, et al., 2018 [62]	Two-center double-blind RCT	100 ICU patients	i.v. 0.2 μg/kg/h Dex from 9:30 pm to 6:15 am vs. placebo, until ICU discharge	- ICU Delirium Screening Checklist every 12 h. - LSEQ	Decrease incidence of ICU delirium (20% Dex group vs. 46% placebo group)	No difference in - sleep quality - incidence of hypotension or bradycardia - More ICU days free of coma in Dex group	- Early mobility was infrequently used - Patients with obstructive sleep apnea were not excluded
Lee H, et al., 2020 [63]	Single-center RCT	217 Postsurgical liver transplant patients	Perioperative i.v. 0.1 μg/kg/h Dex for 48 h vs. placebo	- CAM-ICU every 8 h postoperatively	No difference in delirium incidence (9% Dex group vs. 5.9% placebo group)	No difference in - Delirium duration - Length of MV - ICU LOS - Hospital LOS - In-hospital mortality - 3-month mortality	- Relatively small sample size - Only elective living-donor liver transplant - Unexpected low total delirium incidence in both groups - Probably insufficient Dex’s dose and duration infusion
Turan A, et al., 2020 [64]	Multicenter RCT **(DECADE trial)**	794 cardiac surgery patients	Perioperative infusion of Dex vs. placebo	- CAM-ICU - LSEQ	No significant difference in atrial fibrillation incidence (30% in Dex group vs. 34% in placebo group)	No significant difference in delirium incidence (17% in Dex group vs. 12% in placebo group)	- Subjective delirium assessment - Underestimated delirium incidence
He X, et al., 2021 [65]	Single-center RCT	60 Postsurgical brain-surgical patients	Perioperative i.v. 0.1 μg/kg/h Dex for <24 h vs. placebo	- CAM-ICU every 12 h. postoperatively	Drug interruption rate	No difference in - delirium incidence - bradycardia - hypotension - respiratory depression	- Relatively small sample size - Only brain tumor patients, with preoperative neurological dysfunction - Studies’ primary endpoint was the study-drug interruption
Wang S, et al., 2021 [66]	Systematic Review of 36 studies	9623 ICU patients	Dex vs. non-Dex sedation		Dex was associated with - reduced of delirium risk - higher incidence of bradycardia - and hypotension	Dex was associated with - shorter ICU LOS - shorter hospital LOS - shorter MV duration - No improved mortality	- Low- or very low-quality evidence - Great heterogeneity of included trails
Stangaciu B, et al., 2022 [44]	Single-center Retrospective Clinical Trial	56 MV, severe burn ICU patients, during weaning	Dex 1 mcg/kg bolus and 0.4–0.1 mcg/kg/h iv vs. 0.08 mg/kg/h midazolam or 15–30 mcg/kg/min propofol	- CAM-ICU	- No significantly shorter duration of MV (9.3 vs. 7.5 days), - Lower delirium rate in Dex group (38.4 vs. 53.3%),	- less need for supplemental analgesia (23.1 vs. 53.3%), - less need for antipsychotic agents (15.4 vs. 53.3%) - more incidence of bradycardia in Dex group	- Single-center retrospective study - Limited sample size - Dex patients were younger with higher percentage of 3rd-degree burn injury
Heybati K, et al. [48]	Systematic review of 41 trials	3948 ICU, MV patients	Dex vs. propofol		- No significant difference in ICU LOS - Reduced MV duration - Reduced delirium risk among cardiac surgical patients	- Dex was associated with greater risk of bradycardia, especially among older patients	- Included publications with high risk of bias
Wen J, et al. [50]	Systematic review of 16 RCTs	2035 ICU	Dex vs. midazolam		- Dex achieved shorter ICU LOS - Lower risk of delirium - Shorter duration of MV - More incidence of bradycardia	No difference in - Hypotension - Mortality	- 12 of the 16 trials (n = 1738) explored the incidence of delirium - The studies displayed statistical heterogeneity and small sample size
*2.2 Delirium Treatment*							
Reade MC, et al. [59]	Multicenter, double-blind RCT (DahLIA study)	74 adult intubated ICU patients with agitated delirium	Dex initially at a rate of 0.5 μg/kg/h and then titrated to rates 0–1.5 μg/kg/h vs. placebo	- CAM-ICU - MAAS score	- Increased ventilator-free h at 7 days (144.8 vs. 127.5 h.), - Earlier extubation (21.9 vs. 44.3 h.)	- Accelerated resolution of delirium (23.3 vs. 40.0 h.) - Lower quantities of other sedatives and opioids - Shorter ICU LOS (2.9 vs. 4.1 days) - no difference in incidence of bradycardia, hypotension, or temporarily agitation between the 2 groups	- Relatively small sample size - Different duration of MV before randomization (144.8 h in Dex group vs. 127.5 in placebo)
Lu X, et al. [67]	Single-center double-blind RCT	80 agitated intubated ICU patients	Group A: i.v. 0.3–3 mg/kg/h midazolam for 24 h and then i.v. 0.2–1 μg/kg/h Dex Group B: only i.v. 0.3–3 mg/kg/h midazolam		- HR and MAP were significantly higher in group B during extubation	- Lower incidence of delirium in Dex group (20% vs. 45%) after extubation - Lower extubation time in group A - Lower hospital LOS in group A	- Single-center study - Small sample size - The timing, dosage, and Dex’s target group need to be validated
Serpa Neto A, et al., 2025 [68]	Target-trial emulation	2052 ICU patients’ records with delirium (314 treated with Dex)	Dex vs. other sedatives		- Early initiation of Dex had higher rates of resolution of ICU agitation (94% 30-days delirium resolution vs. 72%)	- Lower risk of tracheostomy by day 30 - Lower mortality (5% vs. 9%)	- Dex patients were younger, had more severe illness, and were more likely to have unplanned department admissions - Potential confounders, such pain level, RASS score, or history of mental illness, medications, or addiction, were not included in the model
Liu X, et al., 2021 [69]	Systematic Review of 10 RCTs and 5 non-RCTs	1017 critically ill patients with delirium	Dex vs. other agents for delirium treatment		- Reduced delirium frequency, - Shorter time of delirium resolution	- Higher rate of bradycardia	- Only a few studies were included in this comparison - High heterogeneity in outcome and interventions
*2.3 Delirium among elderly patients*							
Djaiani G, et al., 2016 [70]	Single-center RCT	183 Cardiac postsurgical patients > 60 years old	i.v. 0.4 μg/kg bolus and 0.2–0.7 μg/kg /h Dex max for 24 h. vs. 25–50 μg/kg/min propofol	- CAM-ICU 12 h postoperatively - CAM when discharged ICU	Decrease incidence of postoperative delirium (17 vs. 31.5%)	- Earlier onset of delirium in propofol group (1st vs. 2nd postoperatively day) - Shorter duration of delirium in Dex group (2 vs. 3 days) - Dex-based sedation was cost savings	- Lack of blinding of drugs infusion - Assessment of CAM and CAM-ICU were not objective - The duration of Dex’s infusion was less than 24 h.
Su X, et al., 2016 [71]	Multicenter, double-blind RCT	700 ICU non-cardiac surgical patients > 65 years old	i.v. 0.1 μg/kg/h Dex vs. placebo	CAM-ICU every 12 h.	Decrease incidence of postoperative delirium (9 vs. 23%)	Hypertension and tachycardia more frequently in placebo (18 vs. 10% and 14 vs. 7%, respectively)	- Lack of baseline delirium assessment, before surgery - CAM-ICU is not a sensitive tool for delirium assessment
Deiner S, et al., 2017 [72]	Multicenter double-blind RCT	404 patients > 65 years old, undergoing non-cardiac surgery	Intraoperative use of i.v. 0.5 μg/kg/h Dex and 2 h postoperatively vs. placebo	- MMSE - CAM every day - CAM-ICU every day	Dex did not significantly reduce postoperative delirium (12.2% vs. 11.4%)		- Daily delirium assessment, during working h, could resalt to undetected evening delirium
Subramaniam B, et al., 2019 [73]	RCT	120 cardiac surgical patients aged > 60 years	Postoperative sedation with propofol vs. Dex and analgesia with acetaminophen vs. placebo	- CAM-ICU every day	- Patients treated with acetaminophen had less delirium incidence than placebo group (10 vs. 28%)	- Patients treated with Dex had no significant difference in delirium rates vs. propofol (17 vs. 21%) - No difference in ICU and hospital LOS	- A relatively small sample size - Possible undetected delirium during evening
Chitnis S, et al., 2022 [43]	Single-center RCT (DIRECT study)	70 postsurgical cardiac patients, >75 years old	Dex vs. propofol	Not referred	Decrease in MCAS score in Dex group	No difference in - delirium rate, - time to extubation, - hospital LOS	- A small open-label single-center study, - Only postsurgical cardiac patients.
Xie K, et al., 2023 [74]	Single-center RCT	236 patients > 60 years old undergoing thoracoabdominal tumor surgery	Postoperative Dex + sufentanil via PCIA vs. only sufentanil via PCIA	Not referred	The incidence of delirium was significant lower in Dex group (3.4 vs. 10.1%)	No difference in ICU and hospital LOS and mortality	- A small single-center study, - Subjective delirium assessment
Huet Q, et al., 2024 [75]	Double-blind RCT	333 postsurgical cardiac patients, >65 years old	Overnight Dex infusion vs. placebo	- CAM-ICU, - LSEQ	The incidence of delirium was not significant different between the two groups (12.6% Dex vs. 12.4% placebo)	Dex group had significantly more hypotensive events (7.3% vs. 0.6%)	- Subjective delirium assessment - Underestimated delirium incidence
Pereira J, et al., 2020 [76]	Systematic Review of 6 RCTs and 2 retrospective cohorts	1249 ICU patients, aged > 60 years	Dex vs. propofol	- CAM-ICU - CAM	Lower delirium risk	No reduced in - ICU LOS, - hospital LOS or - MV duration - No difference in bradycardia or hypotension	- High heterogeneity of included studies and small sample size - High risk of type I and II error
Lin C, et al., 2021 [77]	Systematic Review of 21 studies	Elderly surgical ICU patients with delirium	Dex vs. other sedatives		- Dex reduced delirium incidents in non-cardiac surgical patients, - No difference in delirium frequency among cardiac surgical patients	- Decrease mortality, - Shortened ICU LOS, - Shortened hospital LOS - Increased bradycardia	- Moderate heterogeneity in outcome and interventions
*2.4 Comparative efficacy*							
Carrasco G, et al., 2016 [78]	Single-center non-randomized study	132 non-intubated patients in ICU with agitated delirium	Dex vs. Haloperidol		- Dex achieved higher levels sedation satisfactory - Reduced ICU LOS - Reduced the total ICU cost	Haloperidol associated with - more cases of oversedation - more case of QT prolongation	- single-center study non-RCT
Shokri H, et al., 2020 [79]	Prospective observation RCT	286 cardiac surgery patients >60 years old	i.v. 0.7–1.2 μg/kg/h Dex for 72 h vs. 0.5 μg/kg clonidine	- RASS score	Dex achieved - lower risk of delirium - Shorter MV duration - ICU LOS - lower mortality rate - lower morphine consumption		- single-center study

**Abbreviations:** BDNF: brain-derived neurotrophic factor, CAM-ICU: Confusion Assessment Method for ICU, Dex: dexmedetomidine, h: h., HR: heart rate, ICU: intensive care unit, LOS: length of stay, LSEQ: Leeds Sleep Evaluation Questionnaire, MAAS score: Motor Activity Assessment Scale, MAP: Mean Arterial Pressure, MCAS score: Minnesota Cognitive Acuity Screen score, MMSE: Mini-Mental State Examination, MV: mechanical ventilation, NSE: neuron-specific enolase, PCIA: patient-controlled intravenous analgesia, QALYs: quality-adjusted life years, RCT: randomized controlled trial, S100B: S100 calcium binding protein, SBT: spontaneous breathing trial, UK: United Kingdom.5.4.5. Anxiolytic Effect.

**Table 3:** Clinical indications for dexmedetomidine in ICU patients.

	Study Design	Material	Comparison	Main Outcomes	Study Limitations
3.1. Sleep quality improvement in ICU					
Wu XH, et al., 2016 [95]	RCT	61 non-cardiac postsurgical, non-intubated, ICU patients > 65 years old	Dex 0.1 μg/kg/h vs. placebo for 15 h postsurgically	- Increased the percentage of stage N2 sleep (43.5 vs. 15.8%), - Prolonged the total sleep time, - Decreased the percentage of stage N1 sleep, - Increased the sleep efficiency, - Improved sleep quality - Increased the hypotension incidence	- Single-center pilot RCT - Limited sample size
Lu W, et al., 2017 [94]	Observational Study	20 non-intubated, non-MV, ICU, postsurgical patients	11 in Dex group vs. 9 in no-sedation group	- Sleep efficiency and - Sleep time of patients in the sedation group was significantly higher during the night, - No difference in heart and respiratory rates, - No respiratory depression	- Single-center observational study - Limited sample size
Georgopoulos D, et al., 2021 [91]	Retrospective study	23 MV, ICU patients	Dex vs. propofol vs. no sedation	- In non-sedated patients, sleep quality was poor, with frequent wake intrusions and little stable sleep, - Light sedation with propofol or Dex resulted in a shift in sleep architecture toward normal.	- Small single-center retrospective study
Sun YM, et al., 2022 [96]	RCT	80 non-cardiac, postsurgical, MV, patients	Dex 0.1–0.2 μg/kg/h vs. placebo for >72 h	- Better sleep quality (RCSQ score: 61 vs. 52), - Longer total sleep time, - Higher sleep efficiency, - Lower percentage of stage N1 sleep, - Higher percentage of stage N3 sleep, - Lower arousal index.	- Small sample size - 15% sleep monitoring failure
Qu JZ, et al., 2023 [97]	Single-center RCT (The MINDDS trial)	394 cardiac, postsurgical patients > 60 years old	A short nighttime dose of iv Dex (1 μg/kg in 40 min)	- Non-significant delirium incidence reduction in Dex group (8.8 vs. 14.1%)	- Evaluation of delirium only the first postoperative day - The trial stopped early because of the COVID-19 pandemic
Zhang ZF, et al., 2023 [98]	RCT	118 non-cardiac, postsurgical, non-intubated patients > 65 years old	Dex 0.02 μg/kg/h vs. placebo + opioid analgesia for up to 3 days	- Increased the percentage of stage N2 sleep, - Prolonged total sleep time, - Increased sleep efficiency, - Decrease percentage of N1 sleep, - Lowered sleep fragmentation index	- Single-center RCT - They only monitored sleep quality the night of surgery.
Sun PS, et al., 2024 [99]	RCT	123 non-cardiac, postsurgical, non-intubated patients, with OSA	Dex 0.02 μg/kg/h vs. placebo + opioid analgesia	- Increased the percentage of stage N2 sleep, - Decreased percentage of N1 sleep, - Slightly improved sleep quality	- Single-center RCT - Small sample size
Wang L, et al., 2024 [100]	Systematic review of 29 trials	5610 non-cardiac, postsurgical patients	Perioperative Dex vs. placebo	- Improved sleep quality, - Increased sleep quality,	- Low and very low quality of evidence
3.2. Post-Intensive Care Syndrome					
Zi J, et al., 2020 [90]	Single-center RCT	196 patients underwent off-pump coronary artery bypass	Dex vs. propofol perioperatively	- Lower incidence of atrial fibrillation, - Lower anxiety level (51.6 vs. 67.2%)	- Did not evaluate the effects of other related sedatives or analgesics - Small sample size
Dong CH, et al., 2021 [101]	Single-center RCT	508 patients underwent off-pump coronary artery bypass	Prophylactic nocturnal Dex vs. placebo	- Lower incidence of PICS (21.5 vs. 31.1%), - cognitive impairment (3,98 vs. 6,61%), - disability (2,79 vs. 5,06%), - psychological impairment (18.7 vs. 26.8%), - 6-months mortality (1.2 vs. 1.6%)	- Lack of standard PICS definition - Additional sedatives were used
3.3. Alcohol withdrawal syndrome					
VanderWeide LA, et al., 2016 [102]	Retrospective cohort study	42 AWS ICU patients	Benzodiazepine alone vs. benzodiazepine + Dex	- Reduced benzodiazepine’s requirement, - More cases of bradycardia in Dex group, - No difference in AWS control.	- Small single = center retrospective study - The analysis had bias against Dex
Beg M, et al., 2016 [103]	Retrospective cohort study	77 AWS ICU patients	Benzodiazepine alone vs. benzodiazepine + Dex	- Dex group improved CIWA score, - Dex group increased ICU LOS	- Small single-center retrospective study - The initiation, titration, and discontinuation of the drugs were not control - Additional sedatives were used
Yavarovich ER, et al. [104]	Multicenter retrospective cohort study (8 ICUs)	438 AWS ICU patients	Benzodiazepine alone vs. benzodiazepine + Dex	- Dex group had higher CIWA, - Longer ICU LOS, - Higher rate of delirium.	- Retrospective study - Lack of standardization of Dex prescribing and additional adjunctive medications
Collier TE, et al., 2022 [105]	Single-center retrospective cohort study (The EvADE study)	147 AWS ICU patients	Benzodiazepine alone vs. benzodiazepine + Dex	- No significant change in CIWA-Ar score in Dex group: (3.8 vs. 5.4), - Increased benzodiazepine requirements in Dex group, - Prolonged ICU LOS, - Higher risk of new seizures onset and - Intubation.	- Retrospective study - Lack of standardization of Dex prescribing and additional adjunctive medications - Inability to access CIWA-Ar in intubated patients
Ware LR, et al., 2023 [106]	Single-center retrospective cohort study	197 AWS ICU patients	Phenobarbital alone vs. phenobarbital + Dex	- No-Dex group had reduced ICU LOS (47.5 vs. 97.2 h), - Dex group had higher rates of total delirium days (9208 vs. 143 days), - Dex group had longer MV duration.	- Retrospective cohort
Wong A, et al., 2015 [107]	Review of 13 studies		Dex as an adjunctive agent	- Dex decreased benzodiazepine’s requirement, - Better controlled hypertension and tachycardia, - No difference in seizures control	- Limited number of small trials - Low quality of studies (8 case reports/series and 5 RCTs) - Heterogeneity in AWS assessment and treatment
Woods D, et al., 2015 [108]	Systematic review of 4 studies	55 AWS ICU patients	Dex as adjunctive therapy + standard of care vs. benzodiazepine	- Dex decreased CIWA score, - Decreased delirium more effectively.	- Limited number of small trials (3 retrospective and 1 prospective) - Low-quality studies - Heterogeneity in AWS assessment
Polintan ETT, et al., 2023 [109]	Systematic review of 12 studies	AWS ICU patients	Dex as adjunctive therapy + standard of care vs. benzodiazepine	- Dex showed no significant difference for ICU LOS, - AWS control, - Bradycardia, or - Hypotension.	- Limited number of small trials (3 retrospective and 1 prospective) - Low-quality studies - Heterogeneity in AWS assessment
Fiore M, et al., 2024 [110]	Systematic review of 9 studies (RCTs and non-RCTs)	AWS ICU patients	Dex as adjunctive therapy + standard of care vs. benzodiazepine	- Dex as adjunctive therapy is not more effective than standard of care in reducing intubation, - Higher risk of bradycardia, - Same risk of hypotension.	- Limited number of small trials - Low-quality studies - Heterogeneity in AWS treatment and assessment

**Abbreviations:** AWS: alcohol withdrawal syndrome, CIWA: Clinical Institute Withdrawal Assessment Score, CIWA-Ar: Clinical Institute Withdrawal Assessment Score for alcohol, Dex: dexmedetomidine, h: h., ICU: intensive care unit, LOS: length of stay, MV: mechanical ventilation, OSA: obstructive sleep apnea, PICS: post-intensive care syndrome, RCT: randomized controlled trial, RCSQ: Richards–Campbell Sleep Questionnaire, SBT: spontaneous breathing trial, UK: United Kingdom.

**Table 4:** Neuroprotective effects of dexmedetomidine in ICU patients.

	Study Design	Material	Intervention	Outcomes	Study Limitations
Wu J, et al., 2018 [118]	Animal study	76 male C57 BL/6 mice (TBI model)	Different doses of Dex or placebo in addition, 1 and 12 h after TBI	- Reduced loss of cortical tissue, - Reduced cell death in the cortex and hippocampus - Prevented axonal degeneration - Protected synapses from elimination	Mice treated with Dex were hypothermic
Li F, et al., 2019 [85]	Animal study	Male Sprague-Dawley rats (TBI model)	25 μg/kg Dex 30 min after TBI vs. placebo	- Lower NSS scores - Reduced apoptotic factor expression - Decreased proinflammatory cytokine expression - Increased Nrf2 protein levels - Induced Nrf2 downstream factor expression - Improved TBI induced Bax upregulation and Bcl-2 downregulation	Not refereed
Feng X, et al., 2021 [119]	Animal study	C57 BL/6 mice (TBI model)	30 μg/kg Dex vs. normal saline	- Increased the survival rate and neurological score, - Increased neuron survival, - decreased the expression of the LC3, Beclin-1, and NF-κB proteins, and the cytokines IL-1β, IL-6, and TNF-α	Not refereed
Huang GR, et al., 2021 [120]	Animal study	Rat (BI model)	Dex vs. normal saline	- Inhibit inflammation and - Attenuate early neuronal injury through activation on TLR4/NF-κB pathway.	Not refereed
Liu SY, et al. A 2024 [121]	Retrospective study (Premier dataset)	19,751 ICU patients with TBI	Dex vs. other sedatives	- Reduced hospital mortality - Increased risk for liberation from MV - Reduced LOS in ICU	- Retrospective analysis of MIMIC IV database - Relatively small sample size - Low statistical power
Chen S, et al., 2025 [122]	Retrospective study (MIMIC-IV database)	646 ICU patients with ischemic stroke	Dex vs. other sedatives	- Reduced ICU mortality	- Retrospective analysis of MIMIC IV database
Yang Y, et al., 2025 [123]	Retrospective study (MIMIC-IV database)	2816 ICU patients with ischemic stroke	Dex vs. other sedatives	- Reduced 28-day mortality (27.8 vs. 36.6%) - Reduced 180-day mortality, among discharged patients	- Retrospective analysis of MIMIC IV database - Heterogeneity regarding severity of ischemic stroke
Liu Y, et al., 2025 [124]	Retrospective study (MIMIC-IV database)	527 patients with aSAH	Dex vs. midazolam or propofol	- Reduced in-hospital mortality	- Retrospective analysis of MIMIC IV database - Limited statistical power due to relatively small sample size
Lu S, et al., 2025 [125]	Single-center RCT	60 TBI patients	Dex vs. propofol	- Dex reduced MV duration and ICU LOS - Dex elevated SjvO_2_ and reduced CERO_2_ - Dex reduced IL-6 levels but not IL-1β	- Single-center - Small sample size
Jiang L, et al., 2017 [21]	Systematic review and meta-analysis	19 RCTs including 879 patients	Dex during operation	- Reduces the release of inflammatory mediators and neuroendocrine hormones. - Maintains intracranial homoeostasis - Alleviates ischemic brain injury - exerts an effect on brain protection.	-

Abbreviations: aSAH: aneurysmal subarachnoid hemorrhage, BI: brain injury, Dex: dexmedetomidine, ICU: intensive care unit, IL-6: interleukin-6, INF-γ: interferon-γ, I/R: ischemia/reperfusion, LOS: length of stay, MV: mechanical ventilation, NSS score: Neurological Severity Score System, RCT: randomized controlled trial, TBI: traumatic brain injury, TNF-α: tumor necrosis factor-α.

**Table 5:** Anti-inflammatory effects of dexmedetomidine in ICU patients.

	Study Design	Material	Comparison	Outcomes	Study Limitations
5.1. Cardio-protective properties					
Bulow NMH et al., 2016 [112]	RCT	12 CPB patients in Dex group vs. 11 CPB controls	Dex perioperatively in CPB patients plus other sedatives vs. only other sedatives	- Reduced circulating IL-1, IL-6, TNF-α, and INF-γ levels	- Small sample size - The dose of Dex used was low
Liu X, et al., 2016 [127]	RCT	E60 elective valve and CAP surgery patients	0.2–1.5 μg/kg/h Dex or 5–50 μg/kg/min propofol	- Dex group had significantly greater changes in perfused small-vessel density and the De Backer score	- Possible interaction with other used drugs - Single-center RCT - Small sample size
Yuan B, et al., 2024 [128]	RCT	52 CPB patients vs. 52 controls	0.5 μg/kg Dex before anesthesia induction + 0.5 μg/kg/h vs. 0.125 mL/kg NaCl 0.9% before aortic occlusion	- Serum cTnI, CK-MB, - MDA, and - TNF-α were lower in the Dex group	- Dex group had fewer interventions on the mitral valve
Zhang GR, et al., 2021 [129]	Systematic Review of 17 studies	866 CPB patients	Dex vs. other sedatives	- Reduced serum CK-MB and cTn-I - Shorted ICU LOS - Myocardial protection from I/R injury	- Small sample size - Notable heterogeneity of the included studies
5.2. Renoprotective properties					
Cakir M, et al., 2015 [130]	Animal study	Group 1: 10 I/R injury rats Group 2: 10 controls Group 3: I/R + 10 μg/kg Dex Group 4: I/R + 100 μg/kg Dex	10 μg/kg Dex vs. 100 μg/kg Dex	- Lower levels of MDA, CAT, and GHH in Dex groups - Increased SOD activity in Dex group - In Dex100 group, the elevated BUN levels were significantly lower than in I/R group - In DEX groups, the elevated Cr levels were significantly lower than in I/R group - Lower histological injury scores in Dex groups, but not between different doses of Dex	- It is an experimental laboratory animal study - Only 2 doses of Dex were compared - The efficacy of Dex was evaluated only in kidney I/R injury
Tan F, et al., 2015 [131]	Animal study	Group 1: 8 sepsis model rats Group 2: 8 controls Group 3: 8 Dex + sepsis model Group 4: 8 Dex + yohimbine + sepsis model	10 μg/kg Dex before sepsis vs. a2-adrenergic receptor agonist (yohimbine)	- When rats were pretreated with Dex, sepsis induced AKI was decreased significantly - Exposure of yohimbine eliminated this reduction	- Sepsis AKI is a multifactorial AKI
Zhai M, et al., 2017 [113]	Single-center RCT	72 valve replacement CPB patients	0.6 μg/kg Dex before anesthesia induction + 0.2 μg/kg/h vs. NaCl 0.9%	- The levels of BUN and Cr and the NGAL significantly lower in Dex group, - the intraoperative urine output was significantly increased and - the postoperative incidence of AKI was significantly lower in Dex group.	- AKI was defined according to RIFLE criteria - Single-center small RCT
Liu J, et al., 2020 [132]	RCT	200 MV septic ICU patients	Dex vs. propofol	- Less AKI - Less CRRT duration - Less LOS ICU - No significant difference in MV duration	- Single-center RCT - AKI was defined according to RIFLE criteria
Liu Y, et al., 2018 [133]	Systematic review of 10 studies	1575 postoperative cardiac surgery ICU patients	Dex vs. other sedatives	- Significantly reduced postoperative AKI - No difference in mortality - No difference in MV duration - No difference in ICU LOS and - Hospital LOS	- Relatively small number of RCTs - Small sample size - Great heterogeneity regarding study population or comparison (Dex vs. placebo, or Dex vs. other sedatives) - Different adjuvant sedatives and analgesics
Zhao C, et al., 2024 [134]	Systematic review of 15 RCTs	2907 postoperative cardiac surgical patients	Dex vs. other sedatives	- Dex reduced the incidence of postoperative AKI - No difference in mortality, - No difference in MV duration - No difference in ICU LOS - No difference in hospital LOS	- Moderate study heterogeneity - 4 different AKI definitions - Different RRT initiation criteria
Zhao J, et al., 2024 [135]	Systematic review of 25 trials	3997 postsurgical patients	Dex vs. controls	- Dex reduced the incidence of postoperative AKI - Dex reduced AKI incidence only in cardiac surgical patients - Dex reduced ICU and hospital LOS - Dex reduced postoperative delirium occurrence	- 3 studies focused on pediatric patients - Notably study heterogeneity - Small sample size of non-cardiac surgical patients
5.3. Dexmedetomidine in sepsis					
Al-Regal ARE, et al., 2024 [136]	RCT (The DecatSepsis)	90 Septic ICU patients	Dex infusion for 48 h to maintain the HR:60–90/min	- Underpowered to detect significant reduction in in-hospital mortality and dose of noradrenaline	- Both mechanically and non-MV ICU patients - Small sample size
Iten M, et al., 2025 [137]	Single-Center RCT	70 Septic, MV, ICU patients	0.1–1.4 μg/kg/h Dex vs. propofol/midazolam group	- No difference in S100-β levels at 48 h or upon ICU discharge	- This is a pilot RCT - Small sample size - The duration of Dex sedation was max 7 days - Added propofol or midazolam in Dex group
Patidar AK, et al., 2025 [138]	Single-center RCT	54 septic ICU patients	5 mcg/mL Dex vs. 10 mg/mL propofol, aiming RASS: –2 to 0	- Dex group showed higher mean regional cerebral oxygen saturation - No difference in MV and delirium duration - No difference in ICU LOS	- Single-center RCT - Small sample size
Dargent A, et al., 2025 [58]	The ADRESS Multicenter RCT (5 French ICUs)	Septic shock, MV, ICU patients	Dex vs. placebo in patients with refractory septic shock	- Higher mortality in Dex group - The trial stopped earlier	- Early study elimination
Chen P, et al., 2020 [139]	Systematic Review of 4 studies	349 MV septic patients	Dex vs. other sedatives	- No difference in duration of MV - Significant difference in ventilator-free days and - 28-day mortality	- Limited sample size - Notable study heterogeneity
Wang C, et al., 2021 [140]	Systematic Review of 9 studies	1134 septic ICU patients	Dex vs. other sedatives	- No difference in mortality, LOS ICU, delirium - Significantly reduced MV duration and inflammatory response	- Limited sample size - Notable study heterogeneity
Zhang T, et al., 2022 [141]	Systematic Review of 19 RCTs	1929 septic patients	Dex vs. other sedatives	- Decreased mortality - Decreased inflammatory response - Increased risk of arrhythmias - Not significantly reduce ICU LOS, duration of MV, incidence of delirium, Cr, and ALT	- Relatively small number of RCTs - Limited sample size - Notable study heterogeneity

Abbreviations: AKI: Acute Kidney Injury, ALT: Alanine Aminotransferase, BUN: blood urea nitrogen, CAT activity: catalase activity, CRP: C-reactive protein, Cr: creatinine, CRRT: continues renal replacement therapy, Dex: dexmedetomidine, CK-MB: creatinine-kinase-MB, CPB: cardiopulmonary bypass, Cr: creatinine, cTnI: cardiac troponin I, HR: heart rate, ICU: intensive care unit, IL-6: interleukin-6, INF-γ: interferon-γ, I/R: ischemia/reperfusion, GSH: glutathione, LOS: length of stay, MDA: malondialdehyde, MV: mechanical ventilation, NGAL: Neutrophil Gelatinase-Associated Lipocalin, RCT: randomized controlled trial, RRT: renal replacement therapy, SOD activity: superoxidase dismutase, TNF-α: tumor necrosis factor-α.5.4.9. Neuroprotective Effects.

## Data Availability

Data sharing is not applicable to this article as no new data were created or analyzed in this article.
